# First the relationship, then the technology: Healthcare professionals' perceptions on how digital health solutions impact the interaction with patients

**DOI:** 10.1016/j.pecinn.2025.100448

**Published:** 2025-11-20

**Authors:** S.J. Oudbier, T.A. ten Cate, S.A. Nurmohamed, J.J. Meij, E.M.A. Smets

**Affiliations:** a Amsterdam UMC, Outpatient Division, Amsterdam, the Netherlands; b Amsterdam UMC location University of Amsterdam, Medical Psychology, Amsterdam, the Netherlands; c Amsterdam Public Health Research Institute, Digital Health, Amsterdam, the Netherlands; d Amsterdam Pubic Health research institute, Quality of Care, Amsterdam, the Netherlands; e University Medical Center Utrecht, Faculty of Medicine, Utrecht, the Netherlands; f Amsterdam UMC, Department of Nephrology, Amsterdam, the Netherlands; g Yong Loo Lin School of Medicine, National University of Singapore, Singapore; h Amsterdam UMC, Department of International Affairs, Amsterdam, the Netherlands; i Amsterdam Public Health research institute, Personalized Medicine, Amsterdam, the Netherlands

**Keywords:** Communication, Patient-healthcare provider relationship, Digital health solution, Digital health technology, Video consultation, Home monitoring, Digital care platform

## Abstract

**Objective(s):**

Previous research has explored barriers and facilitators to digital health solutions (DHS) implementation, yet less is known on how DHS should be implemented to maintain patient interaction, and accommodate shifting responsibilities. This study explores health care professionals' (HCP) perspectives on patient-HCP interactions in DHS including video consultations, remote monitoring, and digital care platforms.

**Methods:**

Semi-structured interviews were conducted with twenty-six HCPs. Interviews were transcribed verbatim and thematically analysed by two researchers.

**Results:**

Analysis revealed three overarching themes (the impact of DHS on the relationship, responsibility and anxiety), and ten subthemes. HCPs noted that DHS changed patient-HCP interactions, hindering relationship building. In regard to video consultations, interactional etiquette shifted, with the patient-HCP interaction transitioning towards more informal contact. Remote monitoring and digital care platforms increase patient responsibility, which can hinder communication, particularly when patients access medical results prior to consultations. Most HCPs emphasized that a trusting relationship should be established before introducing DHS to ensure responsible and effective use.

**Conclusion and innovation:**

This study shifts the focus from how DHS can be implemented to how they should be implemented to sustain meaningful patient-HCP interactions. The findings challenge the assumption that DHS can readily replace or supplement traditional care, emphasizing that face-to-face encounters remain essential for patient engagement, relational continuity, and professional fulfilment of HCPs. DHS implementation should therefore follow the establishment of trust and be guided by a tailored, human-centered approach that accounts for individual differences in patients' health status and digital literacy.

## 1 Introduction

In recent years, the use of digital health solutions (DHS) expanded rapidly, with the Coronavirus pandemic of 2019 (Covid-19) serving as a catalyst for widespread implementation [1,2]. DHS refer to the use of digital health technology to improve and optimize the delivery of healthcare [3]. During the Covid-19 pandemic, healthcare professionals (HCPs) had to rely on DHS which enabled remote interaction with patients, such as video consultations, home monitoring systems, and digital care platforms. Video consultations facilitate remote yet real-time, face-to-face communication between patients and HCPs [4]. Home monitoring systems support patients in tracking vital signs and health data within their home environment, promoting self-management and enabling early intervention [5,6]. Lastly, digital care platforms include functionalities such as secure communication between patients and HCPs, access to medical records, and remote monitoring of health metrics [7,8]. These DHS have in common that they allow for interaction with patients, although they vary in functionalities and the level of interaction they facilitate between HCPs and patients. Video consultations facilitate synchronous communication, replicating traditional in-person visits while introducing challenges such as the interpretation of non-verbal cues in a virtual setting [9]. Home monitoring enables asynchronous interactions, wherein HCPs rely on patient-generated health data for clinical decision-making, requiring clearly defined protocols and structured feedback mechanisms to sustain patient engagement and trust [10]. Digital care platforms provide an integrated approach, combining features such as secure messaging, real-time data sharing, and collaborative care planning [8]. By offering greater accessibility, real-time health tracking, and personalized care, these DHS are seen as promising solutions for healthcare challenges such as the ageing population and the increase in healthcare costs [[11], [12], [13]]. Additionally, DHS, specifically those who require some interaction between the HCP and patient, are expected to positively reshape the patient-HCP interaction [14,15]. Indeed, a scoping review demonstrated that DHS could enhance communication, and shift dynamics in the patient-HCP relationship from a hierarchical relationship to one of collaboration [16].

Despite their benefits, implementation of DHS is known to be challenging [17,18]. A recent systematic review showed barriers to the implementation of DHS being well-documented at the organizational level [19]. Common organizational challenges include sufficient training of HCP, adequate allocation of time and resources, and concerns about increasing HCPs' workload [19]. At the same time, this review identified a lack of attention to barriers for implementation at the level of HCPs, despite their essential role as end-users of DHS [19]. Notably, in the few studies addressing HCP’ barriers, potential negative implications for the interaction with patients was ranked among the top three barriers [19].

DHS such as video consultations, remote monitoring, and digital care platforms differ in how they affect interactions between patients and HCPs [20,21]. Prior studies show that while video consultations can reduce personal connection [9,22], remote monitoring may shift responsibilities towards data surveillance [23,24] and digital platforms require new forms of communication and collaboration [14,25]. However, these technologies are often studied in isolation. By examining them side by side, this study identifies both shared and technology-specific dynamics in the patient-HCP interaction that influence their implementation from the perspective of HCPs. Furthermore, HCPs often express varying attitudes towards different DHS, influenced by factors such as familiarity, perceived effectiveness, ease of use, and the specific needs of their patient population [19]. Understanding these attitudes through interviews with HCPs from various departments is essential for tailoring DHS implementation strategies to better meet their needs. Therefore, this study aims to explore HCPs' perspectives on patient-HCP interactions in the context of video consultation, remote monitoring, and digital care platforms.

## 2 Methods

### 2.1 Study design

This is an explorative interview study, to gain in-depth descriptions of HCPs' views towards the adoption and implementation of DHS that have a degree of patient interaction, comprising video consultations, remote monitoring of patients, self-management tools, or interaction via a patient portal. DHS such as artificial intelligence (AI), big data, algorithms, cloud and blockchain were not considered. Semi-structured interviews were conducted with HCPs employed at Amsterdam University Medical Centres, including medical specialists, residents, nurse practitioners, paramedics, and pharmacists, all representing different departments. An interview guide with interview topics was created with a broad scope (Box 1, Supplementary file A), but for this paper the analyses is narrowed down to the impact of the use of DHS on the patient-HCP interaction. The study is reported according to the Consolidated Criteria for Reporting Qualitative Research (COREQ) (Supplementary file B) [26].

**Box 1:** Topics interview guide.

I Introduction to the interview
Short Introduction of the interviewer
Short explanation of the goal of the interview
Explanation of confidentiality and anonymity
Permission for audio recording the interview
II Demographics
III Interview topics
Currently available DHS⁎ that include patient interaction at the HCP's department
Factors influencing DHS implementation
Emphasis on topics such as insecurities, social influence (pressure from colleagues), autonomy and change in HCP⁎-patient relationship
Advantages and disadvantages of DHS implementation
Attention to the use of DHS in continued medical education
IV Conclusion
Future perspectives of HCP on using DHS in the hospital
Remarks and issues that were not yet addressed during the interview

⁎ DHS = Digital Health Solution; HCP = healthcare professional.

### 2.2 Sampling and recruitment

The planned sample size was twenty HCPs, allowing for more participants if needed to reach data saturation. Participants were recruited from Amsterdam University Medical Center by purposive sampling among HCPs (in training) who utilize DHS within as many different disciplines as possible, and varying in age and gender. Firstly, representatives of various disciplines (e.g., surgery, gynaecology, clinical genetics, rehabilitation) were informed about the study and asked to nominate two or three colleagues. Secondly, to not only include individuals with an interest in DHS, participants were asked to nominate a colleague with contradictory beliefs towards the use of DHS. Potential participants were approached via email. Purposeful sampling was used to capture a wide spectrum of perspectives, including those with varying levels of enthusiasm or scepticism towards DHS. To avoid bias in participant selection, we used neutral framing in our invitations; The study was presented as an exploration of experiences and perceptions related to DHS, irrespective of participants' prior interest or familiarity with the subject. Recruitment stopped when data saturation was met, and no new codes (see ‘data analysis’) were identified in at least three subsequent interviews. The initial target of twenty healthcare professionals was expanded to include residents, as their inclusion offered diverse perspectives distinct from other healthcare professionals. Consequently, data saturation was not reached at 20 participants, necessitating further recruitment.

### 2.3 Data collection

The interview guide was created in various rounds with all members of the research team (a psychologist, a medical anthropologist, and two physicians) and using the initial findings of a systematic literature review on barriers and facilitators for the implementation of DHS [19] (Supplementary file A). Two pilot interviews were performed, which resulted in several minimal changes to the structure of the interview guide. The pilot interviews were included in the final data set.

Open-ended questions were used in flexible order (Supplementary file A). Topics of interest were further explored with follow-up questions, until saturation was reached. Fourteen interviews were conducted in a face-to-face setting, the remaining interviews were conducted via the online communication tool Microsoft Teams (Microsoft, Redmond, USA). All interviews were conducted in Dutch by the main researcher (SO, MD, background in medicine). Nine interviews were attended by the second researcher TTC (MD, background in medicine). All interviews were conducted between May 2022 and November 2022. Interviews were audio-recorded after verbal consent, and were transcribed verbatim (SO, TTC). All potentially identifiable characteristics were removed from the transcript to maintain anonymity. After transcription, illustrative quotes were translated into English by a bilingual individual fluent in both English and Dutch to ensure that the original meaning and nuances were accurately preserved.

### 2.4 Ethical considerations

The medical ethical committee of Amsterdam UMC, location VUmc offered a written exemption for the study from the requirement to seek formal approval. We followed the Good Clinical Practice guidelines [27]. Written consent for study participation and publication of results was obtained from all respondents.

### 2.5 Data analysis

Transcripts were analysed and coded in MaxQda (Microsoft, Redmond, Washington, USA) by following the principles of thematic analysis [28]. The two main researchers (SO, TTC) performed the coding. Thematic analysis included reading the transcripts, generating initial codes, and developing and refining themes. The full analysis took place after all the interviews were conducted. Initially, five interviews were coded by both researchers independently and compared for consistency, resulting in a high level of conformity. The coding process was discussed multiple times with one of the senior researchers (ES, psychologist and experienced in qualitative research) and adaptations were made to the codebook. After that, the remaining interviews were coded independently by the two researchers (SO, TTC) who discussed discrepancies until final agreement. Themes were grouped, combined, and refined by both researchers and their content was analysed to generate definitions and labels. Themes were subsequently categorized into overarching themes and subthemes.

## 3 Results

In total, twenty-six HCPs participated in this study: fourteen medical specialists, five residents, and seven other HCPs (nurse, physician assistant, physiotherapist, occupational therapist, pharmacist) (Table 1). Five respondents were recruited who were known for being critical about using DHS. Interviews lasted between 25 and 75 min. The analysis revealed three overarching themes: The impact of DHS on the relationship, responsibility and anxiety, and ten subthemes (see Table 2). Table 3 shows quotes (Q) illustrating the themes.

**Table 1:** Characteristics of the participants (*N* = 26).

Characteristics	n (%)
Sex	
Female	15 (57.7)
Male	11 (42.3)
Age (years)	
25–39	10 (38.5)
40–54	11 (42.3)
55–69	5 (19.2)
Working experience (years)	
1–4	8 (30.8)
5–9	6 (23.1)
10–14	4 (15.4)
≥15	8 (30.8)
Job category	
Medical specialist	14 (53.8)
Resident	5 (19.2)
Other (nurses, physician assistants, occupational therapist pharmacist)	7 (26.9)
Disciplines	
General Medicine	11 (42.3)
Surgical specialties	9 (34.6)
Supportive specialties	6 (23.1)
Interview mode	
Face-to-face	14 (53.8)
Online	12 (46.2)

Data are presented as n(%) unless indicated otherwise.

**Table 2:** Overarching themes, subthemes, and main findings⁎

Theme	Subtheme(s)	Main findings (as experienced by HCPs)
HCP-patient relationship	Concerns about loss of human connection	Video consultations lead to a sense of (emotional) disconnection and difficulty in nonverbal communication
	Less patient interaction	DHS hamper patient interaction thereby interfering with building a good patient-HCP relationship
	Change in conventions, etiquette	Patient interaction dynamics change (formal vs. informal)
	Resentment towards patients due to an increase in workload	Patient contact may decrease as a result of an increase in workload introduced by using DHS
	Professional fulfilment	Job satisfaction declines as the use of DHS is perceived to reduce interpersonal interaction
	Trust	Trust: HCPs prefer face-to-face interactions with patients before implementing DHS to establish trust HCPs prefer face-to-face interaction with patients before implementing DHS to establish trust
	First relationship, then technology	DHS can be employed after establishing a good patient-HCP relationship
Responsibilities	Responsibility HCP	DHS increase patient responsibilities, empowering patients to take charge of their own health. This could complicate effective communication, e.g. patients accessing medical results before consultations
	Responsibility patient	Suitability of DHS varies based on the patient's (health or digital) literacy
Patient anxiety		DHS reduce patient anxiety by providing immediate reassurance through data insights DHS might increase anxiety due to constant monitoring, impacting patient-HCP interaction

⁎ Abbreviations: HCP: Health Care Professional, DHS: Digital Health Solutions.

**Table 3:** Overview of quotes related to overarching themes.

Theme	Subtheme	Quote number (Q)	DHS to which quote related	Characteristics participant	Quote
HCP-patient relationship	Less patient interaction	Q1	Video consultation	Female, surgical specialty, resident	*“Ultimately, I can treat or inform people best when they sit in front of me and I can sense things“*
		Q2	Video consultation	Female, supportive medicine, medical specialist	*“Sitting in the consultation room together sometimes gives you the space to chat about something else than the disease. Sometimes, you just ask in greater detail about someone's profession or you crack a joke. You're more likely to do that in person; it's somewhat more relaxed”.*
		Q3	Video consultation	Female, general medicine, physician assistant	*“To be honest, I'd rather not have such conversations over video call. These are often emotional moments for patients. We talk about life and death. I bought a box of tissues that patients almost always use. In addition, I can position myself towards the patient, turn my chair, sometimes a hand gesture”.*
	Concern that human connection disappears	Q4	Video consultation	Male, general medicine, medical specialist	[said from the perspective of the patient]: “*[..] because I would have preferred to hear it while I was at home… over the phone. That doctor is not my friend, he literally says so. Why do I have to be with him in person? Is it more pleasant for me or more for him? For me it would have been more pleasant if I just heard it at home. Thus, I hear the news and am just home; I do not have to return home, I can simply let out my emotions with my wife next to me..”*
		Q5	Remote monitoring	Male, surgical specialty, medical specialist	“*That is my whole reason for being a surgeon, because I want to make an impact on patients. I can do that even better in ten years [referring to the future use of DHS] than I can do it now, because I am getting to know that patient better. And, and I think that is… That I get closer to the essence of what I want to do”.*
	Change in conventions, etiquette	Q6	Video consultation	Female, supportive medicine, medical specialist	“*I think they don't perceive it as a proper doctor's consultation. Normally when people go see doctor they are right on time, dressed up, and did prepare questions. But yes, we actually experienced differences. Once there was a guy just standing on a ladder, while calling with me. Recently, the doorbell rang during our conversation, because of a fire at the neighbour's appartement”.*
	Increase in workload	Q7	Remote monitoring	Male, general medicine, medical specialist	*“You are buried under things you didn't ask for and patient sometimes also buys those devices [*e.g. *smartwatches]. Without you even knowing it, and then you are sent countless pages of output, and are asked to read it. So well, that is unfeasible, but also causes emotional resistance, because I am already working really hard”.*
		Q8	*	Female, surgical specialty, resident	*“The younger the individuals [HCPs], the more they are willing to make an effort [to familiarize themselves with the technology]. It is noticeable that older individuals often consider it a lost cause. I believe this makes a significant difference”.*
	Professional fulfilment	Q9	Video consultation	Female, supportive medicine	*“If video calls were my only option, I wouldn't like that .. because I personally value the personal contact with patients. Some of that can also be done through video calls, but if I only did that digitally, I wouldn't be happy with it”.*
		Q10	*	Male, general medicine, medical specialist	*“If the goal/pursuit becomes digital, unless… yes, and even worse. If the doctor gets obligations/rules like: You are only allowed to see your patient twice a year, the rest has to be done digitally. If that is dumped on us like a kind of communist assignment, that will harm the patient-provider relationship, but even worse, doctors will no longer enjoy their profession”*
	Trust	Q11	*	Male, general medicine, medical specialist	*“But if you have to undergo major open heart surgery, you just want me to be there. Then you want to sit opposite me and I will have to explain it to you. Then I also have to comfort you and be there for you. And that role will not be taken over by a device, at least not in my lifetime, I think“.*
	First relationship, then technology	Q12	*	Male, general medicine, medical specialist	*You have to see that patient and give them the feeling that there is a person who cares about them… [..] Or afterwards you can support them with anything and everything. I will call you tomorrow or I will video call you tomorrow or I will give you a black smartwatch. But not the other way around”*
	Trust	Q13	Video consultation	Female, general medicine, medical specialist	*There are also patients who do not like it that you literally see them in the living room, [..] It is not always because they have to hide something, but also simply because they do not want to take their privacy to the consultation room”.*
Responsibilities	Patient empowerment	Q14	*	Female, surgical specialty, resident	*I think we should get rid of the idea that the doctor will take care of everything for you. You are responsible for your own health”.*
	Patient empowerment	Q15	Digital care platform	Female, surgical specialty, resident	*Because they can access their patient file, they can already see test results without explanation. Thus, for example, they know they have a malignancy before speaking to a doctor, which causes them to call the hospital over and over again”.*
	Responsibility HCP	Q16	Digital care platform	Male, general medicine, medical specialist	*“If you are ill, [the use of DHS] is highly overestimated, because people do not understand or they are little health literate […] They're just scared and sick and lying in bed sweating asking questions [to the physician, about their disease].”.*
	Responsibility patient	Q17	*	Female, general medicine, nurse	*“People [with cognitive decline] do not always recognize their cognitive limitations, which is why they are in the middle of this process. That is quite difficult, it is not that simple and sometimes I think it is quite a lot to ask”.*
	Responsibility patient	Q18	*	Male, surgical specialty, resident	*“No, we hold ourselves responsible for things that we may not need to be responsible for. You have an excellent alternative for not having to be at the hospital. Thus, you may also expect an effort from the patient to learn how to do that at home and to try that”.*
Patient Anxiety		Q19	Remote monitoring	Male, general medicine, medical specialist	‘*As cardiologists, patients have one thing: fear. And taking that away is also very important and that can be done very well with [DHS]”.*
		Q20	Remote monitoring	Male, general medicine, medical specialist	*“I see 24-year-old men coming to my office because their Apple Watch indicated that their heart rate drops below 40 at night. They come to the office and get an ECHO, a stress test, and… It costs society €400 or €350, while it's just a 24-year-old guy with bradycardia at night. Well, it's nothing really. We all have that”.*
		Q21	Remote monitoring	Female, surgical specialty, medical specialist	“*There are also people who say I do not want to measure my blood pressure every day because it makes me very nervous”.*
		Q22	Digital care platform	Male, surgical specialty, medical specialist	*I could have just had a good conversation about [test results] on Friday. But now I have to call Wednesday, because otherwise they would lie awake in their bed thinking they're dying.*

DHS: Digital Health Solutions. *Quote not related to a specific DHS.

### 3.1 Theme 1: Impact of DHS on HCP-patient relationship

#### 3.1.1 Concerns about loss of human connection

HCPs perceived changes in patient contact following the implementation of DHS. They expressed concerns that using DHS could influence the quality of care (Q1). Some HCPs reported feeling less connected to patients during online consultations, attributing this to reduced non-verbal communication in video consultations compared to face-to-face consultations*.* As a result, HCPs perceived difficulties in building relationships with patients. Moreover, HCPs said that face-to-face interactions allowed more opportunity to discuss topics beyond the patient's illness or to engage in small talk (Q2). In contrast, DHS may positively affect the patient-HCP relationship. One HCP mentioned that video consultations can provide valuable insights into a patient's home environment, behaviours, and interactions, offering a more holistic view of the patient including the presence of support systems such as family members or care givers. A clinical geneticist found video consultations particularly useful, as obtaining family history information often involves distant relatives who are unable to visit the hospital due to geographical or logistical barriers.

#### 3.1.2 Less patient interaction

HCPs commonly responded that DHS are only suitable for certain types of consultations, such as regular outpatient clinic visits. From their experience during the Covid-19 pandemic, HCPs had concluded that delivering bad news is something that should be done face-to-face rather than by video consultation. Some HCPs felt that only face-to-face interaction would allow them to properly intervene when necessary, by steering the conversation or postpone difficult information regarding the disease to a next time, or by attuning to the patient's emotional response. HCPs mentioned that DHS may diminish the crucial aspect of their role: direct, personal interaction with patients (Q3). Interestingly, one HCP suggested to let patients choose whether they prefer a face-to-face consultation or video consultation. This suggestion was based on the HCPs' experience with a patient who had travelled a long distance to the hospital only to receive bad news (Q4).

This HCP proposed that the format of consultations should be tailored to the patient's preferences.

#### 3.1.3 Change in conventions, étiquette

Unlike the majority of HCPs, one HCP mentioned that DHS would bring her into closer contact with a patient. She explained that many of the regular, routine consultations are not useful anyway, such as for chronically ill patients who are stable in their disease. She emphasized that by using DHS, time typically allocated to these routine consultations could be redirected towards patients requiring in-person interactions, thereby allowing her to foster stronger relationships with those patients who benefit most from face-to-face care (Q5). Furthermore, HCPs in our study noted that the use of DHS is mostly beneficial in general medicine as compared to surgical specialties, because in the latter specialty the duration of patient contact is often shorter, with patients typically being referred to their general practitioner or secondary (peripheral) hospital after surgery or treatment. Additionally, HCPs mentioned challenges related to patient behaviours when using DHS, such as patients seemingly taking conversations with physicians less seriously. While patients typically adhere to established conventions during face-to-face interactions with HCPs, some HCPs reported that these conventions were often diminished or even absent in video consultations (Q6).

#### 3.1.4 Resentment towards patients due to an increase in workload

HCPs expressed concerns that the integration of DHS in clinical workflows could increase their workload, particularly through the additional administrative burden, inducing resentment towards the patient (Q7). An HCPs expressed concerns about increased workload due to DHS, as they already experienced limited patient contact, which they feared would decrease further. This reduction could lead to a sense of detachment from patients. In terms of DHS implementation, residents noted that they generally found it easier to adapt to new working methods compared to their more experienced colleagues (Q8).

#### 3.1.5 Reduced professional fulfilment

HCPs repeatedly emphasized the importance of interpersonal interaction for their job satisfaction, and that attention to interpersonal interaction should have a prominent role when implementing DHS. HCPs noted the necessity of balancing digital interactions with face-to-face interactions in delivering healthcare (Q9). One HCP stated this quite firmly (Q10). Notably, this perspective also illustrates how the manner in which DHS are introduced into clinical settings may affect HCPs‘willingness to adopt and integrate these technologies.

#### 3.1.6 The need to establish trust

All of the aforementioned converges in HCPs concerns that ultimately, the human connection with their patients might disappear. HCPs expressed firmly that the HCPs role should not get lost, and that physical proximity is essential. In their opinion, DHS cannot and should not replace direct patient contact (Q11). HCPs indicated that trust is important to consider when introducing DHS. They deem it vital to first invest in a trusting relationship with the patient, before introducing DHS. For example, one HCP felt that complex topics could only be discussed during a video-consultation if a trusting relationship had already been established (Q12). Trust also regards privacy issues. HCPs expressed concerns regarding the growing number of available DHS and the hospital's obligation to implement proper security and privacy standards. They indicated that some patients preferred to not use DHS due to privacy concerns. When using DHS, HCPs claimed that patients expose tend to disclose significantly more personal data than they may intend or feel comfortable sharing (Q13)*.*

### 3.2 Theme 2: Impact of DHS on responsibilities

#### 3.2.1 DHS increase patient responsibilities

HCPs reported how the implementation of new DHS such as home monitoring increased patients' responsibility regarding their own health. Simultaneously, as patients become more self-supporting, for instance, by measuring their blood pressure at home or by doing exercises using a self-management tool, HCPs may have to let go of tasks that before the introduction of DHS were their responsibility. In general, HCPs mentioned that such a shift in responsibilities and empowerment of patients is needed, as they themselves do not have the time to take responsibility for everything that is managed at home (Q14). In contrast, when patients are empowered to take greater control over the management of their own illness, HCPs were concerned that patients could observe everything in their EHR, including results of diagnostic tests. They were particularly concerned about results being available in the EHR, before a scheduled conversation with the HCP. This concern suggests HCPs having reservations about an increase in patient responsibility by having access to their own health information. Some HCPs said information in the EHR was hindering patient contact, as, according to them, patients could not accurately interpret results themselves (Q15). This, in turn, also increased patient anxiety, as patients frequently called the hospital to speak with their HCP for an explanation.

#### 3.2.2 Responsibility patient dependent on the patient's (health or digital) literacy

HCPs indicated that the patient's physical or mental condition determines whether they can handle the responsibility of using DHS. In this context, a distinction can be made between elective (planned) and acute care. HCPs described how, for example, in the case of fertility treatment, the use of DHS (in treatment tracking and monitoring) can provide benefits to the patient. However, in acute care, the situation is different; patients still prefer to have a physician present at their bedside for face-to-face interaction and support: In an emergency, responsibility remains unaltered as HCPs have to take care of the patient (Q16). One HCP mentioned specifically that in case of cognitive decline, no undue responsibility should be placed on the patient (Q17). On the one hand, HCPs felt obliged to first assess whether patients were capable of managing their own health using DHS, before inviting them to do so. Some patients were considered incapable to use DHS due to for example, low health literacy or cognitive impairments. On the other hand, some HCPs argued that in light of necessary changes within the healthcare system, it is important to let go of such responsibilities (to assess whether a patient is suitable or not) and patients should be expected to speak up when support is needed (Q18).

### 3.3 Theme 3: Impact of DHS on patient anxiety

#### 3.3.1 DHS may reduce patient anxiety

HCPs, particularly cardiologists, noted that DHS can help reduce patient anxiety. For instance, providing direct insight to medical data, could immediately reassure a worried patient (Q19). HCPs reported that this enhanced the communication with patients, allowing for quicker intervention in case of patient deterioration.

#### 3.3.2 DHS might increase anxiety

Alternatively, DHS can sometimes cause anxiety in patients as illustrated by one HCP, who observed an increasing trend of young men with bradycardia visiting outpatient clinics, exhibiting heightened anxiety (Q20). HCPs mentioned the importance of identifying which patients are suitable for using DHS. Some patients may experience stress when overwhelmed by excessive health data or when confronted with potentially alarming medical information in their patient file. This in turn would have a negative effect on the interaction with HCPs, because anxious patients are more likely to seek additional consultations (Q21), which could lead to increased time demands, such as extra phone calls for reassurance (Q22).

## 4 Discussion and conclusion

### 4.1 Discussion

Due to an ageing population and a rising number of chronic diseases threatening global healthcare sustainability, DHS are essential to empower patients in self-management and to deliver long-term care outside of hospitals. In this qualitative study, we examined the perspectives of HCPs on the patient-HCP interaction when implementing DHS in healthcare. We found that HCPs characterize implementation of DHS by changes in the HCP-patient relationship, shifting responsibilities, and an impact on patient distress, in particular anxiety. The three types of DHS discussed in this study, video consultation, remote monitoring, and digital care platforms, were each predominantly aligned with a specific theme, although certain elements exhibited overlap. Video consultations, were reported to influence the patient-HCP relationship by enhancing accessibility, although with challenges in maintaining personal connection with a patient. The use of remote monitoring was closely tied to concerns about patient anxiety and consequently led to feelings of ambivalence among clinicians. While monitoring can provide immediate reassurance through accessible data insights, its use may also induce additional stress in patients. The use of digital care platforms was experienced as shifting responsibilities to patients, often leading to empowerment but at the same time raising concerns about patients accessing medical results before consultations, hindering the communication with their HCP.

HCPs generally had the opinion that implementation of video consultations would make them feel more distant from the patient. HCPs described, for example, maintaining eye contact as essential for observing patient behaviour and interpreting non-verbal cues. They considered these cues crucial for providing high-quality care and feared losing these as a result of using DHS. This finding aligns with results from a scoping review that demonstrated how implementation of DHS in general negatively altered patient-HCP dynamics, as using DHS may not fully capture the richness and depth of face-to-face interaction [16]. Additionally, a qualitative study reported HCPs to describe that their patient interactions were notably different when using DHS, lacking human contact and connection [29]. However, the literature also highlights several positive aspects of altered HCP-patient dynamics when using DHS. For example, patients can alert their HCP in case of a deterioration in their condition, which may enhance the patient-HCP relationship, improve patient well-being, and strengthen the communication with HCPs [30,31]. This aligns with our findings. For instance, HCPs noted that patients are more likely to seek medical attention sooner when using DHS, resulting in improved communication with their HCPs.

HCPs in our study said on the one hand that DHS such as video consultations provided opportunities to observe patients' home environments and facilitated the involvement of family members during virtual visits. Consequently, the use of video-consults could improve the provision of patient-centred care by providing insights into a person's lifestyle or behaviours that might not have been addressed during an in-person visit [32]. On the other hand, they reported that during face-to-face clinical encounters, they could better observe patients, and could perform physical examinations which are often needed as they aid in reaching a diagnosis. Despite the increasing reliance on DHS, physical examination remains a crucial aspect of medical practice. Various studies indicate that HCPs' preference for physical examinations is influenced by their medical specialty and what they perceive as critical aspects of the clinical encounter. For example, in ophthalmology, otolaryngology, and orthopaedics, physical examinations are essential for developing a differential diagnosis [33]. Conversely, in specialties such as gastroenterology, nephrology, and endocrinology, the focus is more on the patient's clinical history [33]. Additionally, some studies suggest that physical examination can be conducted remotely, such as in dermatology, where high-resolution smartphone cameras and apps are used for lesion assessment [34,35].

HCPs in our study expressed concerns about shifting responsibilities and patients proactively reading notes and results in the EHR prior to their consultation, potentially leading to misunderstandings. Reading results prior to consultation increased patient anxiety, leading patients to call the outpatient clinic before their consultations to seek explanations from their HCPs. As a result, HCPs experienced frustration, which in turn undermined the relationship with their patients. This is line with other studies showing that remote monitoring can increase patient anxiety due to the generation of error messages and the visibility of abnormal data in the patient portal, hindering the relationship with their HCP [36,37]. Additionally, some patients overuse DHS by measuring their vitals more than necessary [38,39]. HCPs reported that instead of reducing their workload, remote monitoring increased the burden, as they needed to invest more time in reassuring and supporting patients, thereby hindering the relationship. Other studies confirmed this, concluding that overuse of DHS by patients can negatively impact the patient-HCP relationship by diverting the HCPs' attention from their main priority during the consultation [39,40]. This begs the question whether DHS are suitable for patients prone to anxiety. HCPs and patients could discuss the suitability of using a DHS before initiating its use or during an interim evaluation.

Regarding the theme of shifting responsibilities, HCPs in our study stated that they feared delegating too much responsibility to certain patients. They worried that patients with limited health and digital literacy might struggle to effectively use DHS and manage their conditions independently. As a result, HCPs in our study emphasized the importance of first building a trusting HCP-patient relationship before introducing new technology. HCPs could, therefore, assess the patients' capabilities before recommending DHS, and may consider tailored approaches such as 1) providing educational material about DHS that matches the patients' health- and literacy level, such as using step-by-step guides, visual aids, and plain language, 2) providing ongoing technical support, and 3) adjusting usability issues and making sure that DHS are adapted to the patient's needs. Nevertheless, the underlying fear among HCPs that for certain patient populations DHS utilization is not helpful and hindering the patient-HCP interaction may be justified [41]. Significant improvements into the design and implementation of DHS are generally still needed, with a focus on incorporating the needs of both end-users, i.e., patients and HCPs [42].

Interestingly, HCPs' delegation of responsibility to patients for managing their condition seemed to vary depending on the specific disease or condition. This was also confirmed in our study, where HCPs in our study said that DHS are generally more utilized in general medicine specialities than in surgical specialities due to factors such as the duration of the patient contact and the type of disease. Indeed, studies indicate that while DHS can be beneficial for post-operative care by providing remote follow-up and monitoring, its utilization is less frequent in surgical care compared to chronic disease management [43]. However, research also showed that DHS implementation is feasible in surgical specialties, with a multicentre randomised trial showing that women using a personalized DHS for post-recovery after gynaecological surgery was effective in communication with their HCP, and resulted in faster return to work, and higher quality of life [44]. Future research could specifically investigate how different types of communication shape patient-HCP interactions, which would add another layer of understanding to the complex interactions mediated by DHS.

Residents in our study observed how various HCPs differed in familiarizing themselves with DHS. They attributed this variation to differences between generations. Younger generations who have grown up in a digital age, may generally be more comfortable with and therefore more easily embrace new technologies in healthcare [45]. In contrast, older generations may approach DHS more cautiously [46] and may require additional support and training to feel confident in utilizing digital tools in their practice. Residents in our study pointed out that implementation and utilization of DHS are not a standardized component of their curriculum, and emphasized the need to familiarize themselves with (new) DHS, as its use will increase and continue to change the patient-HCP interaction.

#### 4.1.1 Strengths and limitations

Some strengths and limitations of the current study deserve acknowledgement. First, a major strength is our purposive sampling approach, including actively recruiting HCPs who held different perspectives, which resulted in a diverse pool of HCPs. This diversity allows us to uncover interdisciplinary challenges in digital health technology implementation. Second, participating HCPs had experience with various types of DHS, which ensured that the findings do not rely solely on a single DHS. Additionally, the broad initial focus of this study led participants to share their perspectives more spontaneously than if the interview had explicitly concentrated on the topic of HCP-patient interaction. However, this secondary analysis can also be viewed as a limitation, as we may have missed insights because the initial interview guide focused on implementation issues in general, potentially restricting the depth of exploration of the impact on the patient-HCP interaction. While this study provides valuable insights into general perceptions of how DHS influence the patient-HCP relationship, it did not explore the unique effects of specific technologies. For example, the real-time interaction in video consultations may impact patient-HCP relationships differently than the asynchronous interactions of home monitoring or digital care platforms. Future research is needed to investigate these differences to optimally tailor these technologies to specific user needs.

### 4.2 Innovation

This study offers a novel perspective by shifting the focus from the common question of ‘how can we implement DHS?’ to ‘how should DHS be introduced to enhance their interaction with patients’. Unlike many studies that emphasize technical feasibility or patient adoption alone, this research highlights the critical role of relational factors, such as trust-building and personal connection, in the successful implementation of DHS. Key findings from this study challenge the assumption that DHS can easily replace or immediately supplement traditional care. Instead, it emphasizes that DHS should be introduced only after a strong relationship with the patient has been established to ensure better adoption and engagement. The research underscores the importance of face-to-face interactions as foundational for building trust, positioning digital health adoption not merely as a technological challenge, but as a human-centred one. Various stakeholders, including HCPs, hospital managers, professional organizations, and the government, can play a leading role in defining the evolving responsibilities associated with DHS implementation [47,48]. Future research should involve users (patients and HCPs) directly in the design of DHS, by co-creation during the first developmental stages, and by performing a heuristic evaluation combined with think-aloud sessions to identify usability issues already before implementation [49]. DHS are complex systems where two different end users interact asynchronously with the system, adding an extra dimension to the patient-HCP interaction [50]. Following this, process evaluations in implementation research are needed to assess practical deployment and refine DHS for effective use in practice. Despite the potential, many HCPs remain hesitant to adopt DHS, fearing that aspects of their work may take on a different, less rewarding form and that some patients may be hampered rather than helped by the use of DHS. Addressing these concerns through further evaluation and refinement is crucial for ensuring DHS support both HCPs and patients effectively. Finally, the hierarchical nature of traditional medical training and practice can foster a culture of deference to authority and established norms, making HCPs reluctant to deviate from traditional modes of healthcare delivery [51], whereas the introduction of DHS necessitates the development of new competencies among HCPs [29]. HCPs may need to acquire skills in interpreting and analysing patient-generated data, communicating effectively through DHS, ensuring patient privacy, and integrating DHS into clinical practice to improve patient care and health outcomes [52,53].

#### 4.2.1 Practical implications

The findings of this study offer several practical implications for the implementation and design of DHS in clinical settings. For instance, video consultations are particularly well-suited for follow-up consultations where relationships have already been established, but may not be as effective for first-time visits where face-to-face interactions are essential for building rapport and trust. Remote monitoring can improve interaction by allowing HCPs to remotely track patients' health status, facilitating timely interventions and ensuring continuity of care. However, HCPs noted that remote monitoring cannot replace the essential personal with their patients, as the benefits of digital monitoring are best realized when a strong trust relationship is already in place, typically built through face-to-face encounters. Moreover, the successful implementation of remote monitoring relies on the patient's digital literacy and their willingness to adopt new technologies. Digital care platforms, which rely heavily on active patient engagement, may be less effective for individuals with low digital literacy. Therefore, tailored patient education and personal support are crucial to ensure equitable access and optimal use of DHS. These insights highlight the importance of a patient-centred, nuanced approach when integrating DHS into clinical workflows, considering patient characteristics, clinical context, and technological readiness (of both patients and HCPs). Co-creation, involving active participation from both patients and HCPs in the design and implementation of DHS, can ensure that these tools are better suited to the unique needs of individuals, enhancing their relevance and usability [54].

### 4.3 Conclusion

This study revealed that HCPs perceive changes in the dynamics in the patient-HCP interaction as a result of the use of DHS, due to alterations in the patient-HCP relationship, shifting responsibilities, and impact on patient distress. Additional research on the HCP-patient relationship is needed to further explore the changes that occur and to provide guidance for HCPs.

## CRediT authorship contribution statement

**S.J. Oudbier:** Conceptualization, Data curation, Methodology, Formal analysis, Writing - orignal draft, review and editing, Visualization. **T.A. ten Cate:** Conceptualization, Data curation, Methodology, Formal Analysis, Writing - Original draft. **S.A. Nurmohamed:** Writing - review & editing, Supervision. **J.J. Meij:** Conceptualization, Methodology, Writing - review & editing, Supervision. **E.M.A. Smets:** Conceptualization, Methodology, Data analysis, Writing - review and editing, Supervision.

## Declaration of generative AI and AI-assisted technologies in the writing process

During the preparation of this work the author(s) used ChatGPT version 4 in order to translate the interview guide. After using this tool, the author(s) reviewed and edited the content as needed and take(s) full responsibility for the content of the published article.

## Declaration of competing interest

The authors declare that they have no known competing financial interests or personal relationships that could have appeared to influence the work reported in this paper. The primary funder of this project was Amsterdam UMC. There was no external funding available for this project.

## Appendix A Supplementary data

Supplementary material Image 1
