# Supplementary material for: First the relationship, then the technology: Healthcare professionals' perceptions on how digital health solutions impact the interaction with patients
Source: PEC Innov. 2025 Nov 20;8:100448. doi: 10.1016/j.pecinn.2025.100448 (PMC12704259; doi:10.1016/j.pecinn.2025.100448)
Supplement: Supplementary file 1 — Supplementary material [file mmc1.docx]

**Appendix**

**Supplementary file A.** Interview guide

**Introduction**

- Introduce ourselves.
- May I record this interview? The recording will be used for research purposes and will be processed anonymously. The data will be deleted after 10 years.
- All information will be treated confidentially:
  - Regarding privacy: Data will be stored on a sound recorder, then transferred to the secure AMC drive (G-drive). The data will be deleted from the recorder afterward. It will be transcribed, anonymized, and analyzed (it will no longer be traceable to the individual). The data will be deleted no later than 10 years after the study.
- Do you have any questions before we begin?

**General Information**

- Name
- Age
- Gender
- Specialty
- Number of years in the field

**Ice Breaker**

- What comes to mind when you hear the term "new technology"? What technologies do you use in your daily life? (Definition of wearable: a small electronic device worn close to the body that monitors vital signs such as oxygen levels, blood pressure, and heart rate, without restricting mobility. If necessary, ask the participant to specify which technology is the main focus of the conversation.)

**Introduction to Why I Became a Doctor – REFLECTIVE INTRODUCTION**

- What motivated you to become a doctor or work in healthcare?

**Technology in Healthcare**

- What are your thoughts on the current technological developments in your work? (Consider technological advancements such as video calls, home monitoring, wearables, etc.)
- What do you see as the benefits of these new technologies?
- What do you perceive as the drawbacks of these new technologies? (e.g., impact on autonomy)

**Factors Affecting Technology Implementation**

- We’ve just discussed your views on new technology in healthcare. Regarding the implementation of these technologies, what are your personal barriers to adopting new technologies in the workplace?
- What factors contribute to the successful use of new technologies in the workplace?
- What can you do to support the implementation of new technology? What is most important in this regard?
- What is needed to keep you engaged in the implementation of technology?
- What are the barriers for others (nurses, outpatient staff) to implementing technology?
- What is needed to keep your colleagues involved in the implementation of new technology?
- What kind of training or other measures are necessary to make technologies accessible and maintain their accessibility? (Focus on the type of technology mentioned > what kind of technology is it and is it relevant?)

**Future Perspective**

- We have discussed implementation. Lastly, I would like to talk about the retention of technology and its future.
- Can you provide an example of a technology that you started using but eventually stopped or gave up? Can you explain why this happened?
- If you continue to use it, what factors contributed to that?
- What are your expectations for future technologies in healthcare?
  - For example, break it down by various technologies: what are your expectations for how these technologies will change healthcare in the coming years?
- Reflecting on why you became a doctor/professional in healthcare, does technology bring you closer to your goal, further away, or does it make no difference? Can you explain? (autonomy)

**Additional Questions for Residents**

- What role does digital innovation play in your training as a specialist?
- Has digital innovation, and the resulting changes in the way you work as a specialist, influenced your choice of specialization?
- To what extent are you, as a resident, involved in the implementation of new technologies?
- Do you see a difference between residents and specialists regarding the implementation of new technologies? If so, what do you think causes this difference?

**Closing**

- Do you have any additional comments or anything you would like to add?
- Do you have any final questions?
- Thank the interviewee.

(*translated from Dutch to English with ChatGPT version 4, 2024).

**Supplementary file B.** COREQ guidelines.
